# Does Real-World Evidence of the Economic Burden of Lung Cancer in Greece Exist? A Systematic Review of the Literature

**DOI:** 10.3390/curroncol32030130

**Published:** 2025-02-25

**Authors:** George Gourzoulidis, Catherine Kastanioti, George Mavridoglou, Theodore Kotsilieris, Dikaios Voudigaris, Charalampos Tzanetakos

**Affiliations:** 1 Department of Business Administration and Organizations, School of Management, University of the Peloponnese, 24100 Kalamata, Greece; a.kastanioti@go.uop.gr (C.K.); t.kotsilieris@uop.gr (T.K.); 2 Department of Accounting and Finance, School of Management, University of the Peloponnese, 24100 Kalamata, Greece; ge.mavridoglou@go.uop.gr; 3 Health Through Evidence GP, 17456 Athens, Greece; d.voudigaris@hte.gr (D.V.); c.tzanetakos@hte.gr (C.T.)

**Keywords:** lung cancer, economic burden, real-world evidence, Greece

## Abstract

Objective: This systematic literature review aimed to summarize the economic burden of lung cancer in Greece, identify current data gaps, and support the design of future real-world studies. Methods: A systematic search of studies published in English on the cost of lung cancer was performed in MEDLINE-(PubMed), Scopus, and ScienceDirect. The databases were searched until September 2024, and records were screened based on our eligibility criteria. After conducting the initial literature search, the abstracts and full texts of the identified studies were reviewed and evaluated for inclusion based on predefined criteria. Data from the selected studies were then extracted into a standardized form and subsequently synthesized. Results: Seven studies were included in this review. The reported burden was sourced from hospital data and categorized as direct and indirect costs. Most studies (n = 6) reported direct costs, with one study reporting both direct and indirect costs. The total direct medical cost per patient increased from approximately EUR 16,000 in 2015 to EUR 58,974 in 2023, with drug acquisition costs being the key driver of the total direct cost. Additionally, the cost of end-of-life care during the final six months of a patient’s life was estimated to range from EUR 6786 to EUR 7665 per patient, with pharmaceutical costs comprising the largest proportion of the total cost. One study also reported that indirect costs were considerably higher for patients than for family caregivers. Conclusion: The economic burden of lung cancer has increased substantially over the past decade in Greece. The present systematic review emphasizes the critical need for comprehensive real-world studies on the economic burden of lung cancer in Greece. Addressing the current gaps holistically will yield invaluable insights for policymakers and stakeholders.

## 1. Introduction

Lung cancer is the most commonly diagnosed cancer worldwide and remains the leading cause of cancer-related mortality [1]. In Greece, lung cancer is the third most commonly diagnosed cancer overall (first for males and fourth for females) and the leading cause of cancer-related deaths. According to 2020 estimates, 6640 new cases were diagnosed in males, accounting for 74% of total cases, and 2320 cases in females [1]. Similarly, lung-cancer-related deaths totaled 5940 in males (77% of all deaths) and 1722 in females. This evidence highlights a significant gender disparity in lung cancer incidence and mortality, emphasizing the need for targeted strategies to address gender-specific disease burdens [1].

Lung cancer is broadly classified into two main types: non-small cell lung cancer (NSCLC) and small cell lung cancer (SCLC) [2]. NSCLC accounts for 80–90% of lung cancer cases and includes three primary histological subtypes: adenocarcinoma, which represents approximately 40% of cases; squamous cell carcinoma (SCC), comprising 25–30% of cases; and large cell carcinoma, which constitutes 10–15% [3].

In addition to its clinical impact [4,5,6], lung cancer places a substantial economic burden on healthcare systems across Europe [7,8]. The high direct costs associated with the management of advanced NSCLC patients are primarily driven by hospitalizations [9,10,11], drug administration [10,11], and the treatment of adverse events (AEs) [11]. These costs increase with disease progression compared to stable disease [12,13,14]. As most patients with NSCLC are of a working age, this can lead to detrimental effects on employment due to impaired productivity [15,16] and absence [15,16,17,18] and impose a substantial financial burden on patients, their caregivers, and the economy.

A mix of public and private sector involvement characterizes the Greek healthcare system. The National Health System (NHS) provides universal healthcare coverage, primarily financed through taxation and social insurance contributions [19]. Within the NHS, healthcare services, including hospital care and pharmaceuticals, are largely reimbursed by the state, ensuring accessibility to essential treatments. The private healthcare sector, on the other hand, plays a complementary role, catering to patients seeking additional services or shorter waiting times [19]. Reimbursement policies for high-cost treatments, such as those for lung cancer, are guided by the Ministry of Health and the Health Technology Assessment Committee [20].

However, in Greece, real-world evidence regarding lung cancer is limited, with only a few studies being available. Therefore, conducting a comprehensive and structured analysis of the existing research is crucial to clearly understand lung cancer’s financial impact on the NHS and society. In this context, the objective of the current study was to conduct a systematic literature review to investigate the economic burden of lung cancer in Greece. Additionally, the study aimed to identify data gaps and provide insights to support the design of future real-world studies.

## 2. Materials and Methods

This systematic review followed the guidelines of the Preferred Reporting Items for Systematic Reviews and Meta-Analyses (PRISMA) 2020 [21]. The current systematic literature review (SLR) is registered in the International Prospective Register of Systematic Reviews (PROSPERO) under the registration number CRD42024623682.

### 2.1. Search Strategy

A systematic search for studies published in English on the cost of lung cancer in Greece was conducted using electronic databases. More specifically, the Scopus and ScienceDirect databases were searched using the following keywords and Medical Subject Headings terms: (economic OR cost OR healthcare cost OR cost analysis OR expenditure) AND (lung cancer OR lung neoplasm) AND (Greece OR Hellas). For PubMed, a different set of keywords was used, as it allows for more precise and structured search capabilities compared to other databases. Table S1 in the Supplementary Material provides the complete search strategy for PubMed. The search included no restrictions on the original studies’ time frame or geographical location. However, the search was confined to studies published up to September 2024. In addition, the reference lists of all relevant articles that were initially selected for inclusion in the review and related reviews were manually searched in Google Scholar to identify any potentially relevant articles that may have been missed during the initial electronic search.

### 2.2. Study Selection

Following the literature search, duplicate studies were removed, and the remaining studies were independently screened by two reviewers using predetermined inclusion criteria based on the PICOS framework (Population, Interventions, Comparators, Outcomes, and Study Design). Details of the PICOS criteria that were applied in the search strategy are outlined in Table 1. Any disagreements between the reviewers regarding the eligibility of specific studies were resolved through discussion with a third reviewer. Modeling studies such as cost-effectiveness analyses were excluded from the review, as the focus was on real-world evidence data. The study selection process was divided into two stages. In the first stage, studies were assessed based on their titles and abstracts to determine whether they met the eligibility criteria. In the second stage, full-text articles were retrieved for further screening if titles and abstracts lacked sufficient information or suggested that the studies met the inclusion criteria. A study was excluded if there was insufficient information to make an inclusion decision after reviewing the full text. The entire selection process was documented using a flowchart to track the number of studies at each stage.

### 2.3. Data Extraction and Synthesis

Data extraction was performed using a standardized form, developed specifically for this review. Two reviewers independently extracted the data; a third reviewer was consulted to resolve discrepancies. The extraction form was designed to record key details, including authors, publication year, study design, year of data collection, sample size, study perspective, and the cost outcomes reported in each study. The economic burden of lung cancer was analyzed by categorizing costs into direct and indirect components.

Moreover, this systematic review presents the findings qualitatively and quantitatively by compiling data from various studies. The relevant and available data were systematically synthesized in alignment with the review question and the established inclusion and exclusion criteria. Additionally, given that some studies did not calculate the cost per patient, the mean cost per patient was calculated by dividing the total estimated costs by the study’s sample size. When studies reported costs in currencies other than euros, a conversion was performed using a cost converter tool provided by the Campbell and Cochrane Economics Methods Group and the Evidence for Policy and Practice Information and Co-ordinating Centre [22].

### 2.4. Quality Assessment of Included Studies

The quality of the included studies was assessed using a modified version of the Effective Public Health Practice Project (EPHPP) Quality Assessment Tool [23]. The methodological dimensions evaluated included allocation bias, study design, confounders, blinding, data collection methods, attrition bias, intervention integrity, and statistical analysis. Each domain was rated on a three-point scale (strong, moderate, weak) based on predefined criteria and guidelines for tool application. An overall quality rating was assigned to each study: studies with no weak ratings were classified as strong, those with one weak rating as moderate, and those with two or more weak ratings across the six domains as weak.

## 3. Results

### 3.1. Search Results and Characteristics of Included Studies

**Figure 1:**
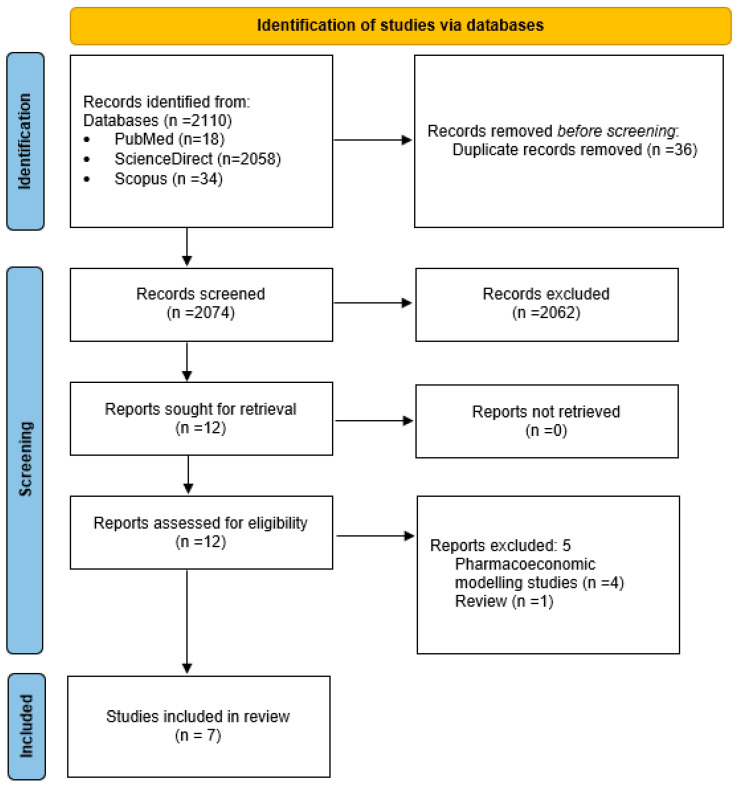
PRISMA 2020 flow diagram of systematic review.

The search process identified 2110 studies related to the topic of the review. Of these, 2098 were excluded based on a screening of their titles and/or abstracts, leaving 12 studies for full-text review. Seven [24,25,26,27,28,29,30] studies met the selection criteria and were included in the final review. The PRISMA 2020 flowchart (Figure 1) illustrates a detailed breakdown of the literature search strategy, outlining the number of studies that were assessed at each stage of the review process.

The included studies were published between 2001 [26] and 2023 [24], covering study durations ranging from six months [26] to five years [25]. Notably, 88% of the studies were conducted over a time horizon of one year or more. Two [24,25] of the included studies were multicentric or nationwide, while the remaining five were conducted in single-center settings [26,27,28,29,30]. Most studies (five out of seven) employed retrospective designs [24,25,27,29,30], while all used a bottom-up costing approach. Furthermore, all studies were conducted from a Greek public payer perspective, except for one study that also considered a societal perspective [28] (Table 2).

The sample sizes of the studies ranged from 30 to 346 patients, with 60% of the studies having a sample size of more than 100 patients. Most studies focused on populations of patients with NSCLC or SCLC, often in the advanced stages of the disease. At the same time, treatment approaches varied, ranging from end-of-life care and chemotherapy to novel therapies and surgical interventions (Table 2).

All the included studies [24,25,26,27,28,29,30] reported direct costs, and one study also provided data on direct and indirect costs [28]. Common cost drivers included drug acquisition, hospitalization, and diagnostic testing. One study also assessed indirect costs, such as productivity losses for patients and caregivers. Two studies [24,25] received funding, while the remaining five either did not report external funding or explicitly stated that no funding was received [26,27,28,29,30] (Table 2).

### 3.2. Direct and Indirect Medical Costs

Direct medical costs encompass immediate healthcare expenses related to patient care, such as hospitalizations, drug acquisition, diagnostic testing, and physician ser-vices. Across the included studies, drug acquisition costs and hospital-related expenses constituted the largest proportion of direct medical expenditures.

A study by Leftakis and Geitona [26] reported a detailed analysis of hospitalization costs for thoracic surgical patients with lung cancer who were admitted to the intensive care unit (ICU) of Sotiria Hospital in Athens, Greece, between September 1997 and February 1998. This prospective analysis of 95 patients who underwent surgery and required intensive postoperative care estimated an average total hospitalization cost of EUR 6057 per patient, with ICU costs accounting for 29% of this total (Table 3). This study highlighted a notable discrepancy between the costs that were reimbursed by public health insurance and the actual costs incurred by the hospital.

The study by Gkogkozotou et al. [29] focused on evaluating PET/CT and brain MRI in the staging of NSCLC in the oncology unit from December 2014 until November 2016. The study included 30 patients, divided into three groups: Group 1 (surgery after PET/CT), Group 2 (surgery after neoadjuvant chemotherapy), and Group 3 (chemotherapy without surgery). The cost components analyzed included diagnostic test costs, nonsurgical treatment costs, and surgical treatment costs. The average diagnostic test cost (including PET/CT and brain MRI) for patients in Group 3 was EUR 1823, while surgical treatment costs were significantly higher, averaging EUR 9068 for Group 2. The total costs per group were EUR 7701 (Group 1), EUR 9476 (Group 2), and EUR 5109 (Group 3) (Table 3). Notably, the study highlighted that PET/CT significantly improved the staging accuracy, detecting metastases that were missed by CT in 10% of cases. This resulted in avoiding unnecessary surgeries for some patients and improving the prognoses for others by redirecting them to neoadjuvant chemotherapy before surgery, thereby enhancing the imaging outcomes and patient care.

**Figure 2:**
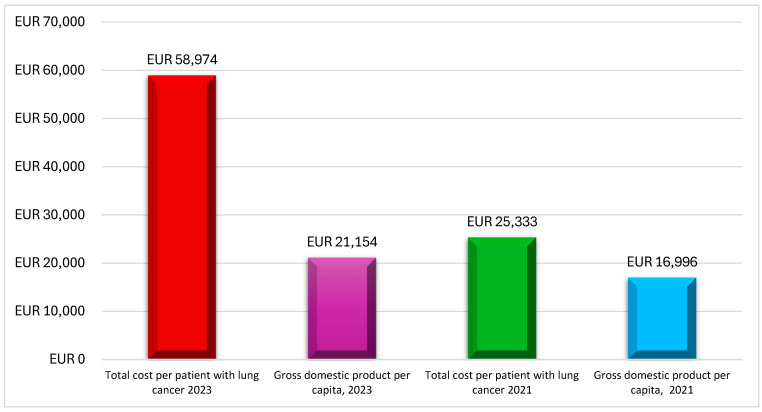
Lung cancer per patient costs compared with Greek GDP per capita. Source: This figure is the authors’ creation based on studies by Mountzios et al. (2021) [25] and Linardou et al. (2023) [24], as well as International Monetary Fund (IMF) data on Greece [31].

Mountzios et al. [25] provided real-world evidence for 59 patients with advanced NSCLC harboring epidermal growth factor receptor (EGFR) mutations, who were treated between 2015 and 2020. The study estimated the total direct cost per patient to be EUR 25,334, with EUR 21,865 being attributed to drug acquisition, EUR 3325 to monitoring, and EUR 143 to managing severe AEs (Table 3). Notably, this total direct cost per patient (EUR 25,334) exceeded Greece’s 2021 Gross Domestic Product (GDP) per capita (EUR 17,000), as reported by the International Monetary Fund (IMF), indicating the significant economic impact of advanced therapies like afatinib (Figure 2).

Linardou et al. [24] investigated the real-world costs of NSCLC patients who were previously treated with immunotherapy. This study included 346 patients from 18 clinical centers across Greece, making it one of the largest of its kind in the country. The mean public payer expenditure per patient was EUR 58,974, with EUR 58,008 for drug acquisition costs, EUR 570 for monitoring, EUR 203 for administration, and EUR 192 for managing Grade 3–4 AEs (Table 3). This cost was calculated considering that each patient remained on nivolumab treatment for an average of 27.53 treatment cycles. This cost was nearly three times Greece’s 2023 GDP per capita, highlighting the substantial financial burden associated with immunotherapy (Figure 2).

Zarogoulidou et al. [28] analyzed both the direct and indirect costs of managing lung cancer patients in Greece, focusing on 113 patients who were diagnosed with either NSCLC or SCLC at the Pulmonary Department of the University of Thessaloniki. The total direct costs amounted to EUR 1,853,984, with chemotherapy drugs representing the largest expense (EUR 1,216,421, or 70%). Other significant costs included supportive care (EUR 147,373) and hospitalization (EUR 85,308). The average direct cost per patient, calculated by dividing the total costs by the number of patients, was approximately EUR 16,407 (Table 3). The study also explored the indirect costs, which amounted to 28,774 lost days of productivity, of which the majority (95%) were attributed to patients, with caregivers accounting for the remaining 5%. Interestingly, no significant relationship was found between indirect costs and patient factors like gender, smoking status, or stage of disease, underscoring the universal burden of productivity loss among lung cancer patients. While the study did not find a clear link between the quality of life and the costs incurred, it did conclude that the burden of lung cancer management on healthcare systems remains substantial, particularly due to the growing use of high-cost chemotherapy drugs.

The studies by Souliotis et al. [27] and Kokkotou et al. [30] provided comprehensive analyses of the economic burden associated with end-of-life care for lung cancer patients, drawing on real-world data from Sotiria Hospital in Athens, Greece. Souliotis et al. [27] examined healthcare expenditures for 144 Stage IIIB/IV lung cancer patients during their last six months of life, reporting a total cost of EUR 977,310 (averaging approximately EUR 6786 per patient), with inpatient services (57%) and chemotherapy (42%) being the main contributors (Table 3). This study highlighted the economic impact of intensive hospital-based interventions and aggressive chemotherapy regimens.

In contrast, Kokkotou et al. [30] reported a mean per-patient cost of EUR 7665 for the last six months of life, with 75% being attributed to pharmaceuticals and 16.2% to radiation therapy (Table 3). The study found a marginally significant difference in costs based on cancer type, with adenocarcinoma patients incurring higher median costs (EUR 9031) compared to SCC (EUR 6606) and SCLC (EUR 5474) patients.

Both studies highlight the substantial economic burden associated with pharmaceutical interventions during end-of-life care. Despite the limited life expectancy of terminal patients, the persistent use of high-cost therapies raises critical questions about balancing clinical benefits and economic impacts.

### 3.3. Results of Quality Assessment of Included Studies

The quality of the included studies was assessed using a modified version of the EPHPP (Effective Public Health Practice Project) tool, revealing significant variation in methodological rigor. The results of the quality assessment of the included studies are summarized in Table 4. More specifically, the studies by Linardou et al. (2023) [24], Gkogkozotou et al. (2018) [29], and Zarogoulidou et al. (2015) [28] achieved strong overall ratings, demonstrating robustness in study design, data collection, and intervention integrity. Conversely, moderate ratings were observed in the studies by Souliotis et al. (2019) [27], Kokkotou et al. (2021) [30], Mountzios et al. (2021) [25], and Leftakis et al. (2001) [26], primarily due to limitations in addressing confounders and blinding.

## 4. Discussion

The current systematic review identified seven studies that examined the economic burden of lung cancer in Greece, offering valuable insights and revealing substantial gaps in the existing evidence base. The included studies varied in their design, scope, and methodology. Five of the studies [24,25,27,29,30] were retrospective, while two were prospective [26,28], and most were conducted in single-center settings, with only two adopting a multicenter approach. The sample sizes ranged from 30 to 346 patients, and the majority focused on advanced or terminal-stage NSCLC or SCLC. Direct medical costs were a predominant focus across all studies [24,25,26,27,28,29,30], with only one study addressing direct and indirect costs [28].

Although the reviewed studies were not directly comparable due to differences in design, the findings revealed a significant increase in the direct costs per patient over the past decade, primarily driven by the introduction of advanced therapies such as targeted treatments and immunotherapies. Specifically, the total direct costs per patient have risen from approximately EUR 16,000 in 2015 [28] to EUR 58,974 in 2023 [24], driven primarily by drug acquisition expenses. This trend mirrors broader global patterns in oncology, where the costs of novel therapies substantially exceed those of traditional chemotherapy.

End-of-life care also represents a considerable economic burden [27,30]. Costs during the last six months of life were predominantly attributed to pharmaceuticals, particularly in patients with adenocarcinoma. These findings align with prior studies in Europe and globally, emphasizing the disproportionate financial impact of aggressive pharmaceutical interventions during terminal stages.

Indirect costs, such as productivity losses for patients and caregivers, were only assessed in one study [28]. This underscores a critical gap in the literature, as the societal impact of lung cancer extends beyond direct medical expenses. Comprehensive analyses that include both direct and indirect costs are essential to provide a holistic understanding of the disease’s economic burden.

Our results align with prior SLRs that have examined the economic burden of lung cancer. For instance, an SLR conducted in China [32] highlighted the substantial impact of direct medical costs, including drug acquisition and hospitalizations, similar to the findings from Greece. However, the study also identified significant data gaps, particularly in indirect cost analyses—a challenge that is mirrored in the Greek context. Additionally, SLRs on SCLC identified chemotherapy and diagnostics as key cost drivers [33], which is consistent with the Greek studies, where drug acquisition predominates. Another SLR on early-stage NSCLC underscored escalating costs as the disease progresses, complementing the Greek evidence, which shows a substantial rise in economic burden due to advanced therapies in later stages [34].

The management of lung cancer in Greece is a critical challenge for the NHS. Real-world evidence (RWE) studies play a pivotal role in addressing this issue by providing a comprehensive understanding of the economic burden of lung cancer. These studies capture the true costs of treatment, encompassing hospitalization, medication, and productivity losses, offering valuable insights into the financial strain on the Greek healthcare system. Such insights are essential for effective healthcare planning, enabling policymakers to allocate resources efficiently, evaluate the cost-effectiveness, and develop targeted interventions to reduce clinical and financial burdens. By bridging the gap between theoretical economic models and real-world practice, these studies provide a grounded perspective on the challenges that are unique to Greece.

Addressing existing knowledge gaps includes an urgent need for a robust RWE study in Greece. This study should employ a comprehensive framework that accounts for both direct and indirect costs to fully capture the economic impact of lung cancer. Furthermore, it should include a diverse and representative patient population, reflecting the heterogeneity of lung cancer across different stages, histological subtypes, and treatment strategies. Such an initiative would offer a clearer and more accurate assessment of the economic burden of lung cancer and support the development of cost-effective, targeted strategies to alleviate this burden. Insights generated from this research could guide policymakers in optimizing healthcare resource utilization and ensuring the sustainability of the healthcare system

As with all related studies, our review has certain limitations. One notable limitation is the potential for publication bias, as only studies published in English were included in this review. In addition, the search was limited to free databases. Moreover, the methodological heterogeneity observed across the included studies also poses challenges for synthesis and interpretation. Most studies relied on retrospective data from single-center settings, which may not capture the diversity of patient populations and healthcare practices across Greece, which limits the generalizability of findings to the broader population. In contrast, other SLRs [32,33,34,35] often include multicenter and longitudinal studies that provide a more representative picture of cost trends and resource utilization. Furthermore, while the EPHPP Quality Assessment Tool is widely recognized for its robust evaluation across key methodological domains, it may not fully capture the nuanced differences between study designs, such as retrospective versus prospective studies.

## 5. Conclusions

The economic burden of lung cancer in Greece has grown substantially, driven by rising drug costs and the increasing adoption of advanced therapies. Addressing current data gaps through comprehensive RWE studies will provide critical insights for policymakers, enabling more informed decisions on healthcare resource allocation. A multifaceted approach that balances clinical effectiveness with economic sustainability is essential to reduce the burden of lung cancer on patients, caregivers, and the healthcare system

## Figures and Tables

**Table 1:** Study selection criteria considered in the search strategy.

	Inclusion Criteria	Exclusion Criteria
Population	Lung Cancer	-
Interventions	-	-
Comparators	-	-
Outcomes	Original studies investigating direct or indirect costs of lung cancer	-
Study design/type	Prospective, retrospective, observational studies (published full paper of original study)	Reviews or meta-analyses Editorials Comments Letters to the Editor Presentations at scientific conferences Pharmacoeconomic modeling studies
Language restrictions	English language publications	-
Date of publication	Studies published until September 2024	-
Country	Greece	Countries other than Greece

**Table 2:** Characteristics of included studies.

Study	Study Design and Data Source	Population and Sample Size	Disease Stage	Study Duration	Treatment Status at Study Inception	Costing Method	Perspective of Analysis	Funding
Leftakis et al. (2001) [26]	Prospective cohort study; hospital patient records (Athens)	95, patients with lung cancer treated in the ICU	All stages undergoing surgery	1997–1998 (6 months)	Postoperative patients after thoracic surgery	Bottom-up	Public payer perspective (Greek NHS)	Not reported
Zarogoulidou et al. (2015) [28]	Prospective cohort study; hospital patient records (Thessaloniki)	113, patients diagnosed with NSCLC or SCLC	Local and extended disease (NSCLC/SCLC)	2011–2014 (32 months)	Newly diagnosed patients undergoing chemotherapy	Bottom-up	Public payer and societal perspective	None
Gkogkozotou et al. (2018) [29]	Retrospective cohort study; hospital patient records (Athens)	30, patients diagnosed with NSCLC	Early and advanced stages (I-IV)	2014–2016 (2 years)	Patients undergoing surgery or chemotherapy treatments	Bottom-up	Public payer perspective (Greek NHS)	None
Souliotis et al. (2019) [27]	Retrospective cohort study; hospital patients records (Athens)	144, patients with terminal-stage NSCLC or SCLC	Stage III B/IV	2011–2014 (3 years)	Lung cancer patients at end-of-life stage	Bottom-up	Public payer perspective (Greek NHS)	None
Mountzios et al. (2021) [25]	Retrospective cohort study; patient records (clinical centers across Greece)	59, patients diagnosed with EGFR mutation-positive NSCLC	Locally advanced or metastatic (Stage III/IV)	2015–2020 (5 years)	Patients treated with afatinib (1st-line, 2nd-line, or beyond)	Bottom-up	Public payer perspective (Greek NHS)	Hellenic Cooperative Oncology Group internal research grant
Kokkotou et al. (2021) [30]	Retrospective cohort study; hospital patient records (Athens)	122, patients with terminal-stage NSCLC or SCLC	Stage IV	2015–2018 (4 years)	Lung cancer patients at end-of-life stage	Bottom-up	Public payer perspective (Greek NHS)	None
Linardou et al. (2023) [24]	Retrospective cohort study; patient medical records (clinical centers across Greece)	346, patients diagnosed with advanced NSCLC	Stage IV	2015–2019 (4 years)	Patients previously treated with other therapies, starting nivolumab	Bottom-up	Public payer perspective (Greek NHS)	Hellenic Cooperative Oncology Group

NSCLC: non-small cell lung cancer; SCLC: small cell lung cancer. EGFR+: epidermal growth factor receptor-positive.

**Table 3:** Reported costs of lung cancer from the included studies.

Study	Disease Stage	Direct Cost per Patient (EUR)	Key Cost Drivers	Indirect Cost
Leftakis et al. (2001) [26]. ±	All stages undergoing surgery	6057	ICU costs accounted for 29% of total costs.	NR
Zarogoulidou et al. (2015) [28] *	Local and extended disease (NSCLC/SCLC)	16,407	Drug acquisition (chemotherapy drugs) accounted for 70% of the total cost, followed by supportive care (8%) and hospitalization (5%).	28,774 missed workdays
Gkogkozotou et al. (2018) [29]	Early and advanced stages (I–IV)	7701 (Group 1) 9476 (Group 2) 5109 (Group 3)	Diagnostic tests (EUR 1649–EUR 1823), surgical treatment (EUR 4416–EUR 9068), and nonsurgical treatment (EUR 1523–EUR 2227).	NR
Souliotis et al. (2019) [27] *	Stage III B/IV	6786	A total of 57% of costs were associated with inpatient services, and 42% were driven by chemotherapy drugs.	NR
Mountzios et al. (2021) [25]	Locally advanced or metastatic (Stage III/IV EGFR+)	25,334	Drug acquisition (EUR 21,865, 86%), monitoring (EUR 3325, 13%), and AEs (EUR 143, 1%).	NR
Kokkotou et al. (2021) [30]	Stage IV	7665	Drug acquisition (EUR 5749, 75%), followed by radiation therapy (EUR 1241, 16%), laboratory/imaging tests, and hospitalization cost (EUR 639, 8%).	NR
Linardou et al. (2023) [24]	Stage IV	58,974	Drug acquisition (EUR 58,008, 98%), monitoring (EUR 570, 1%), and administration and AEs (EUR 395, 1%).	NR

NSCLC: non-small cell lung cancer; SCLC: small cell lung cancer; EGFR+: epidermal growth factor receptor-positive; adverse events: AEs; ICU: intensive care unit; ± costs in Leftakis et al. (2001) [26] were originally reported in US dollars and converted to euros for consistency. * For studies that did not report the cost per patient directly, the mean cost per patient was calculated by dividing the total reported costs by the sample size.

**Table 4:** Quality assessment of included studies.

Study	Allocation Bias	Study Design	Confounders	Blinding	Data Collection Methods	Attrition Bias	Intervention Integrity	Statistics	Overall Rating
Souliotis et al. (2019) [27]	√	√	√	Not mentioned	√	√	√	√	Moderate
Kokkotou et al. (2021) [30]	√	√	√	Not mentioned	√	√	√	√	Moderate
Mountzios et al. (2021 [25])	√	√	√	Not mentioned	√	√	√	√	Moderate
Zarogoulidou et al. (2015) [28]	√	√	√	√	√	√	√	√	Strong
Leftakis et al. (2001) [26]	√	√	√	Not mentioned	√	√	√	√	Moderate
Linardou et al. (2023) [24]	√	√	√	√	√	√	√	√	Strong
Gkogkozotou et al. (2018) [29]	√	√	√	√	√	√	√	√	Strong

## Data Availability

All input data for the study are available in the tables published in this manuscript.

## Supplementary Materials

The following supporting information can be downloaded at https://www.mdpi.com/article/10.3390/curroncol32030130/s1, Table S1: Full search strategy used for PubMed. Reference [21] is cited in the supplementary file.
