# Supplementary material for: Does Real-World Evidence of the Economic Burden of Lung Cancer in Greece Exist? A Systematic Review of the Literature
Source: Curr Oncol. 2025 Feb 25;32(3):130. doi: 10.3390/curroncol32030130 (PMC11941143; doi:10.3390/curroncol32030130)
Supplement: Supplementary file 1 [file curroncol-32-00130-s001.zip › curroncol-3393723-supplementary.pdf]

Supplementary Material

Table 1: Full search strategy used for PubMed

(((((((((((direct cost[Title/Abstract]) OR (indirect cost[Title/Abstract])) OR (economic[Title/Abstract])) OR (cost[Title/Abstract])) OR (healthcare cost[Title/Abstract])) OR (resource utilization[Title/Abstract])) OR (resource use[Title/Abstract])) OR (cost analysis[Title/Abstract])) OR (treatment cost[Title/Abstract])) OR (expenditure[Title/Abstract]))) AND (lung cancer[Title/Abstract])) AND (Greece[Title/Abstract])
